# A late presenting congenital diaphragmatic hernia misdiagnosed as spontaneous pneumothorax

**DOI:** 10.4103/0019-5049.71034

**Published:** 2010

**Authors:** Chitra Sanjeev Juwarkar, Deependra Suresh Kamble, Vishal Sawant

**Affiliations:** Department of Anaesthesia, Goa Medical College, Goa, India; 1 Department of Paediatric Surgery, Goa Medical College, Goa, India

**Keywords:** Chest tube, congenital diaphragmatic hernia, pneumothorax

## Abstract

Congenital diaphragmatic hernia (CDH) is described as (1) failure of diaphragmatic closure at development, (2) presence of herniated abdominal contents into chest and (3) pulmonary hypoplasia. Usually, pleural space is drained urgently when there is respiratory distress and radiological appearance of mediastinal shift. We present a case of a 5-month-old baby, diagnosed as tension pneumothorax and treated with chest drain insertion. CDH was the intraoperative diagnosis.

## INTRODUCTION

Congenital diaphragmatic hernia (CDH) is herniation of bowels and abdominal solid organs in thoracic cavity, causing pulmonary hypoplasia with decreased pulmonary vasculature and dysfunction of the surfactant system. In severe cases, left ventricular hypoplasia is also observed.

Infants have scaphoid abdomen, barrel-shaped chest and respiratory distress (retractions, cyanosis, grunting respirations). In 1848, Bochdalek first described a CDH case which resulted from developmental failure of posterolateral foramina to fuse properly.[1] Incidence of CDH is 1:2000 to 1:5000 with equal gender preference.[2] Incidence of herniation is 78–90% posterolaterally through foramen of Bochdalek, 1.5–6% retrosternally via foramen of Morgagni and 14–24% via oesophageal hiatus. Left-sided herniation is common, as right hemidiaphragm develops earlier and the liver prevents hollow viscus from herniation.[3] On the affected side, there is right to left shunting of blood. Also, 70–95% cases are diagnosed in the neonatal period; later diagnosis becomes difficult due to vague cardiorespiratory and gastrointestinal symptoms.[3] Emergency correction is required in most cases.

Usually, plain chest radiograph done after passing a nasogastric tube gives the diagnosis of CDH. However, it may be misinterpreted and diagnosed as pleural effusion/pneumothorax (as in our case), and hence, there is a possible risk of chest tube perforating herniated viscus.[4] The anaesthetic management of our case was no different from that of an early diagnosed neonatal diaphragmatic hernia.

## CASE REPORT

A 7-kg, 5-month-old, cyanotic male baby was referred to us as a case of spontaneous pneumothorax with respiratory distress unresponsive to medical management.

On examination, the baby was afebrile, irritable, cyanosed, with a heart rate of 146/minute, tachypnoeic with a respiratory rate of 78/minute, with nasal flaring, subcostal and intercostal retraction and grunting. The chest expansion on the left side was decreased and there was a hyper-resonant note with absent air entry. The right lung had decreased air entry with rales and conducted sounds and trachea was deviated to the right. SpO_2_ on room air varied between 70 and 80%. Cardiovascular and gastrointestinal system showed no gross anomaly.

Investigations revealed the following: haemoglobin 8.3 g%, Random Blood Sugar level (RBSL) 193 mg%; Arterial Blood Gas (ABG) (FiO_2_ 34% approx.) revealed pH 7.468, PCO_2_ 25.5, PO_2_ 81.1, SaO_2_ 96.9, HCO_3_ 18.6, Base Excess (BE) –2.7.

**Figure 1:**
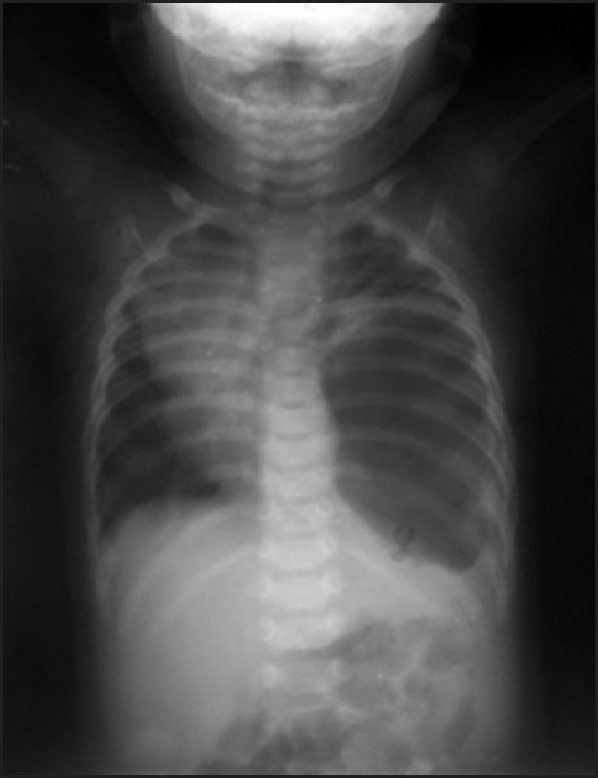
Plain chest X-ray masquerading as a tension pneumothorax on the left side with mediastinal shift on the right side

**Figure 2:**
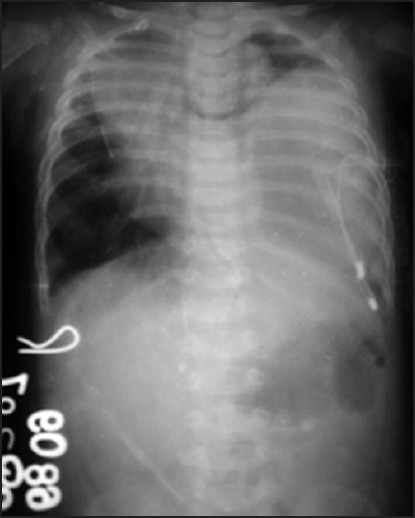
Same chest X-ray after ICT insertion

Chest X-ray showed massive, left-sided pneumothorax with mediastinal shift to the right side [Figure 1]. Earlier attempts to drain pneumothorax had failed, and repeat chest X-ray was suggestive of haemopneumothorax, with three Intercostal Chest Tubes (ICTs) introduced on the left side [Figure 2]. A computed tomography (CT) scan revealed a localised area of air pocket, which was reported as encysted pneumothorax or possibly congenital lobar emphysema.

Paediatric surgeon posted the baby for emergency thoracotomy.

### Anaesthetic management

The baby [American Society of Anaesthesiologists (ASA) grade III E] was shifted to Operation Theatre, wrapped in cotton wool gamgees in a radiant warmer, receiving O_2_ by hood and a vein secured with 24-g i.v. cannula. Resuscitative drugs/airway cart were kept ready. ECG, pulse oximeter and neonatal BP cuff were attached. The baby was premedicated with i.v. atropine 0.14 mg, dexamethasone 2.5 mg and ondansetron 1 mg.

After preoxygenating the baby with 100% oxygen by mask using Jackson Rees’ (JR) circuit for 5 minutes, ketamine 14 mg was administered i.v. and halothane in 100% oxygen delivered in increments from 0.5 to 3% maintaining spontaneous ventilation. During induction, SpO_2_ gradually started dropping; hence, tracheal intubation was immediately done with size 4 uncuffed oral endotracheal tube. After confirming optimum air entry on both the sides, the tube was fixed and the neonate was allowed to breathe spontaneously. Ventilation was then assisted as saturation started improving. Anaesthesia was now maintained with 100% oxygen and 1% halothane with assisted ventilation using JR circuit.

**Figure 3:**
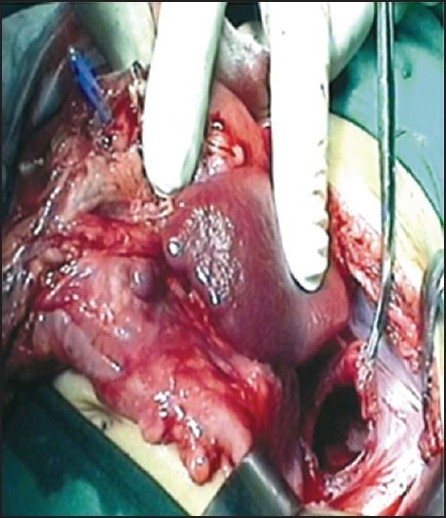
Intraoperative view showing diaphragmatic defect

The baby was placed in a right lateral position for a left thoracotomy incision. Surprisingly, bowel loops popped out of left thoracotomy incision, pointing to a diagnosis of CDH. The baby was immediately made supine to proceed for laparotomy [Figure 3]. Neuromuscular blockade was then achieved with i.v. pancuronium 0.7 mg and the ventilation controlled. On withdrawing the bowel loops from thoracic cavity, both oxygen saturation and air entry improved remarkably. Analgesia was provided with i.v. pethidine 3.5 mg.

ECG, BP and SpO_2_ were monitored intraoperatively. Also, i.v. fluids were given as per Holliday Segar formula using Isolyte-P (50 ml/hour; maintenance fluid + operative loss), and blood loss (approx. 50 ml) was replaced with fresh whole blood. At the end of operation, i.v. ondansetron 1 mg and dexamethasone 2.5 mg was repeated. Residual muscle blockade was reversed with i.v. neostigmine 0.35 mg and i.v. atropine 0.14 mg. Once respiratory efforts were adequate, the baby was extubated after thorough oral and tracheal suctioning.

Postoperatively, there was improved air entry and SpO_2_ was maintained at 96% on room air. The baby was shifted to intensive care unit on a mobile warmer with O_2_ administered via polymask. Postoperative chest X-ray showed good bilateral lung expansion, and chest drains were then removed after 72 hours. Rest of the postoperative period was uneventful. The baby was discharged after 7 days.

## DISCUSSION

Bush *et al*.[5] as well as a recent publication in *Hong Kong Journal of Paediatrics*[6] described a case of CDH misdiagnosed as pneumothorax, resulting in ICT insertion. Hence, investigations like chest X-ray (with or without nasogastric tube insertion), contrast studies, CT scan and laparoscopy are to be done to exclude diaphragmatic herniation.[7]

Our case was similar to this with the only difference being presentation at 5 months after birth which probably led to the mistaken diagnosis. The initial fall in SpO_2_ and the subsequent improvement following reduction of contents of hernia sac are self-explanatory.

Initially, reduction of herniated viscera and diaphragmatic defect closure were emergently performed following birth. With the recent understanding that pulmonary hypoplasia and hypertension determine the outcome of CDH,[8] surgical intervention has been delayed enabling preoperative stabilisation of the patient’s clinical condition. Failure to thrive requires gastrostomy tube placement to improve the caloric intake. The goal of CDH management is lung expansion avoiding over distension; therefore, inspiratory pressures should be lowest. High frequency ventilation lowers the ventilator pressures and normalises PaCO_2_. Frequent radiography helps in assessment of lung expansion. Extra Corporeal Membrane Oxygenation (ECMO) is used for very sick infants, but is available at fewer centres.

Face mask ventilation and nitrous oxide cause gastric insufflation, further impairing circulation/respiration, and hence, they are avoided. We used only 100% O_2_ as SpO_2_ dropped below 90% and we avoided N_2_O (facility to provide air lacked in our anaesthesia machines). In theory, positive pressure ventilation preferentially ventilates normal lung. At the same time any re-expansion of collapsed lung will exacerbate the mass effect, thereby worsening the circulation. Collapsed lung should therefore be isolated and normal lung ventilated with small tidal volumes/pressures using a double lumen tube, until decompression of affected hemithorax is achieved. At least single lumen tube with bronchial blocker should be considered.[9] We have no experience of one lung ventilation technique with double lumen tubes in our institute.

Caudal epidural anaesthesia is good, but is rarely opted in case of emergencies. Introduction of epidural nerve stimulation and ultrasound guidance for inserting epidural catheter up to mid-thoracic segments have been described.[10]

## References

[1] Rout S, Foo FJ, Hayden JD, Guthrie A, Smith AM (2007). Right-sided Bochdalek hernia obstructing in an adult: Case report and review of the literature. Hernia.

[2] Bhardwaj M, Taxak S, Rattan KN, Goyal P, Aggarwal M (2008). Late presentation of congenital diaphragmatic hernia-anaesthetic considerations. Internet J Anesthesiol.

[3] Robinson PD, Fitzgerald DA (2007). Congenital diaphragmatic hernia. Paediatr Respir Rev.

[4] Gupta S, Raiger LK, Shah D, Kumar S (2005). Late presentation of congenital bochdalek hernia. Indian J Anaesth.

[5] Coren ME, Rosenthal M, Bush A (1997). Congenital diaphragmatic hernia misdiagnosed as tension pnemothorax. Pediatr Pulmonol.

[6] Tsui KP, Chan KW, Lee KH (2010). Acute complications of late-presenting congenital diaphragmatic hernia in children. HK J Paediatr (New Series).

[7] Cigdem MK, Onen A, Otcu S, Okur H (2007). Late presentation of Bochdalek-type congenital diaphragmatic hernia in children: A 23 year experience at a single center. Surg Today.

[8] Rohana J, Boo NY, Thambidorai CR (2008). Early outcome of congenital diaphragmatic hernia in a Malaysian tertiary centre. Singapore Med J.

[9] Shah R, Reddy AS, Dhende NP (2007). Video assisted thoracic surgery in children. J Minim Access Surg.

[10] Tsui BC (2006). Innovative approaches to neuraxial blockade in children: The introduction of epidural nerve root stimulation and ultrasound guidance for epidural catheter placement. Pain Res Manag.

